# Regulation of actin-binding protein ANLN by antitumor *miR-217* inhibits cancer cell aggressiveness in pancreatic ductal adenocarcinoma

**DOI:** 10.18632/oncotarget.18261

**Published:** 2017-05-29

**Authors:** Tetsuya Idichi, Naohiko Seki, Hiroshi Kurahara, Keiichi Yonemori, Yusaku Osako, Takayuki Arai, Atsushi Okato, Yoshiaki Kita, Takaaki Arigami, Yuko Mataki, Yuko Kijima, Kosei Maemura, Shoji Natsugoe

**Affiliations:** ^1^ Department of Digestive Surgery, Breast and Thyroid Surgery, Graduate School of Medical Sciences, Kagoshima University, Kagoshima, Japan; ^2^ Department of Functional Genomics, Chiba University Graduate School of Medicine, Chiba, Japan

**Keywords:** microRNA, miR-217, pancreatic ductal adenocarcinoma, ANLN, tumor-suppressor

## Abstract

Analysis of our microRNA (miRNA) expression signature of pancreatic ductal adenocarcinoma (PDAC) revealed that *microRNA-217* (*miR-217*) was significantly reduced in cancer tissues. The aim of this study was to investigate the antitumor roles of *miR-217* in PDAC cells and to identify *miR-217*-mediated molecular pathways involved in PDAC aggressiveness. The expression levels of *miR-217* were significantly reduced in PDAC clinical specimens. Ectopic expression of *miR-217* significantly suppressed cancer cell migration and invasion. Transcription of actin-binding protein Anillin (coded by *ANLN*) was detected by our *in silico* and gene expression analyses. Moreover, luciferase reporter assays showed that *ANLN* was a direct target of *miR-217* in PDAC cells. Overexpression of *ANLN* was detected in PDAC clinical specimens by real-time PCR methods and immunohistochemistry. Interestingly, Kaplan–Meier survival curves showed that high expression of *ANLN* predicted shorter survival in patients with PDAC by TCGA database analysis. Silencing *ANLN* expression markedly inhibited cancer cell migration and invasion capabilities of PDAC cell lines. We further investigated *ANLN*-mediated downstream pathways in PDAC cells. “Focal adhesion” and “Regulation of actin binding protein” were identified as *ANLN*-modulated downstream pathways in PDAC cells. Identification of antitumor *miR-217*/*ANLN*-mediated PDAC pathways will provide new insights into the potential mechanisms underlying the aggressive course of PDAC.

## INTRODUCTION

Due to the aggressive nature of pancreatic ductal adenocarcinoma (PDAC), it is one of the most lethal malignancies in the world [1, 2]. Recently developed targeted molecular strategies have not contributed to improved therapies for PDAC, and the 5-year survival rate after diagnosis is only 5% [2, 3]. More than 50% of patients develop local recurrence or distant metastasis after curative resection. PDAC frequently metastasizes to the liver, peritoneum and lung [4, 5]. We hypothesized that current genomic approaches might be used to elucidate the molecular mechanisms underlying PDAC metastasis and suggest improved treatments for this disease.

microRNA (miRNA) belongs to a family of small non-coding RNAs that fine-tune the expression of protein coding/noncoding RNAs by repressing translation or cleaving RNA transcripts in a sequence-dependent manner [6]. A unique characteristic of human miRNAs is that a single miRNA species can regulate a large number of RNA transcripts [7, 8]. Therefore, dysregulated miRNAs can disrupt tightly controlled RNA networks and promote cancer cell metastasis. At present, numerous studies have indicated that miRNAs are aberrantly expressed in several cancers, including PDAC [9–11]. The discovery of miRNAs and subsequent studies have deepened our understanding of the roles of miRNA in human cancer pathogenesis [12, 13].

Using miRNA expression signatures, we have identified antitumor miRNAs that modulate novel cancer networks in several types of cancer [14–16]. More recently, we reported the anti-tumor function of *miR-375*, and the observation that it regulated oncogenic zinc finger protein 36 ring finger protein-like 2 (*ZFP36L2*) in PDAC cells [17]. Moreover, *ZFP36L2* was overexpressed in PDAC specimens and Kaplan–Meier survival curves showed that high expression of *ZFP36L2* predicted shorter survival in PDAC patients [17]. We have also constructed an miRNA expression signature of PDAC clinical specimens based on RNA-sequencing. Our data showed that *miR-217* was significantly downregulated in PDAC tissues, suggesting that it functions as an antitumor miRNA that targeted several oncogenic genes in PDAC cells. Past studies demonstrated that *miR-217* acts as an antitumor miRNA in several types of cancer [18–20]. *miR-217* expression was observed to be negatively correlated with KRAS protein expression in PDAC cell lines and *miR-217* directly regulated *KRAS* [21, 22]. However, the RNA networks mediated by *miR-217* in PDAC are still obscure.

The aim of this study was to investigate the antitumor roles of *miR-217* in PDAC cells and to identify *miR-217*-mediated molecular pathways involved in PDAC aggressiveness. Our present data showed that the actin-binding protein anillin (ANLN) (coded by the *ANLN* gene) was directly regulated by antitumor *miR-217* in PDAC cells. Kaplan–Meier survival curves showed that high expression of *ANLN* predicted shorter survival in patients. Moreover, we showed that “Focal adhesion” and “Regulation of actin binding protein” were downstream pathways modulated by ANLN protein in PDAC cells. Elucidation of antitumor *miR-217*-mediated molecular networks in PDAC may provide new insights into the potential mechanisms of PDAC aggressiveness.

## RESULTS

### Expression levels of *miR-217* in PDAC specimens and cell lines

**Figure 1:**
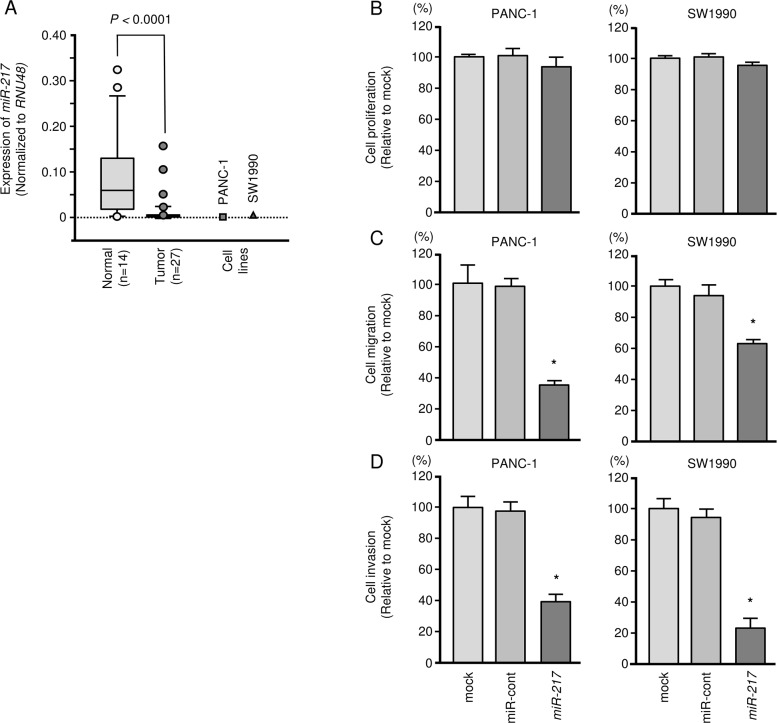
Antitumor functions of *miR-217* in PDAC cell lines (PANC-1 and SW1990) **(A)** Expression levels of *miR-217* in PDAC clinical specimens and cell lines were determined by qRT-PCR. Data were normalized to *RNU48* expression. **(B)** Cell proliferation was determined by XTT assays 72 h after transfection with 10 nM *miR-217*. *, *P* < 0.0001. **(C)** Cell migration activity was determined by migration assays. *, *P* < 0.0001. **(D)** Cell invasion activity was determined using Matrigel invasion assays. *, *P* < 0.0001.

We evaluated expression levels of *miR-217* in PDAC tissues (n = 27), normal pancreatic tissues (n = 14) and two PDAC cell lines (PANC-1 and SW1990). The clinical samples’ backgrounds and clinicopathological characteristics are summarized in Table 1A. Normal pancreatic tissues are summarized in Table 1B. The expression levels of *miR-217* were significantly lower in tumor tissues compared with normal pancreatic tissues (*P* < 0.0001, Figure 1A, Supplementary Figure 1). However, there were no significant relationships between any of the clinicopathological parameters, (i.e., neoadjuvant chemotherapy, metastasis or recurrence) and the expression of *miR-217* (data not shown).

**Table 1A:** Patient characteristics

Pancreatic ductal adenocarcinoma (PDAC)			(%)
Total number		27	
Average age (range), years		67.1 (42-85)	
Gender	Male	12	(44.4)
	Female	15	(55.6)
T category	pTis	1	(3.7)
	pT1	2	(7.4)
	pT2	0	(0)
	pT3	22	(81.5)
	pT4	2	(7.4)
N category	0	15	(55.6)
	1	12	(44.4)
M category	0	25	(92.6)
	1	2	(7.4)
Neoadjuvant chemotherapy	(−)	12	(44.4)
	(+)	15	(56.6)
Recurrence	(−)	7	(25.9)
	(+)	20	(74.1)

**Table 1B:** Patient characteristics

Normal pancreatic tissue			
Total number		14	
Average age (range), years		63.8 (42-85)	
Gender	Male	6	(42.8)
	Female	8	(57.2)

### Effect of *miR-217* expression on cell growth, migration and invasiveness in PDAC cell lines

To investigate the functional roles of *miR-217*, we performed gain-of-function studies using transfection of PANC-1 and SW1990 cells. The expression levels of *miR-217* were markedly lower in two cell lines (Figure 1A). To elucidate molecular mechanisms of low expression of *miR-217* in PDAC cells, PANC-1 and SW1990 cells were treated with the demethylating agent [5-aza-2′-deoxycytidine (5-aza-dC)]. Expression levels of *miR-217* in PDAC cells were significantly elevated by 5-aza-dc treatment (Supplementary Figure 2). These data suggested that DNA methylation might cause silencing of *miR-217* in PDAC cells.

XTT assays revealed no significant inhibition of cell proliferation in PANC-1 or in SW1990 cells transfected with *miR-217* in comparison with mock or control transfectants (Figure 1B). *In vitro* assays demonstrated that migration and invasion were significantly inhibited in *miR-217* transfectants compared with mock or miR-control transfectants (each, *P* < 0.0001, Figure 1C and 1D, Supplementary Figure 5). These results suggested that *miR-217* could have an antitumor function in PDAC cells.

### Identification of candidate genes regulated by *miR-217* in PDAC cells

**Figure 2:**
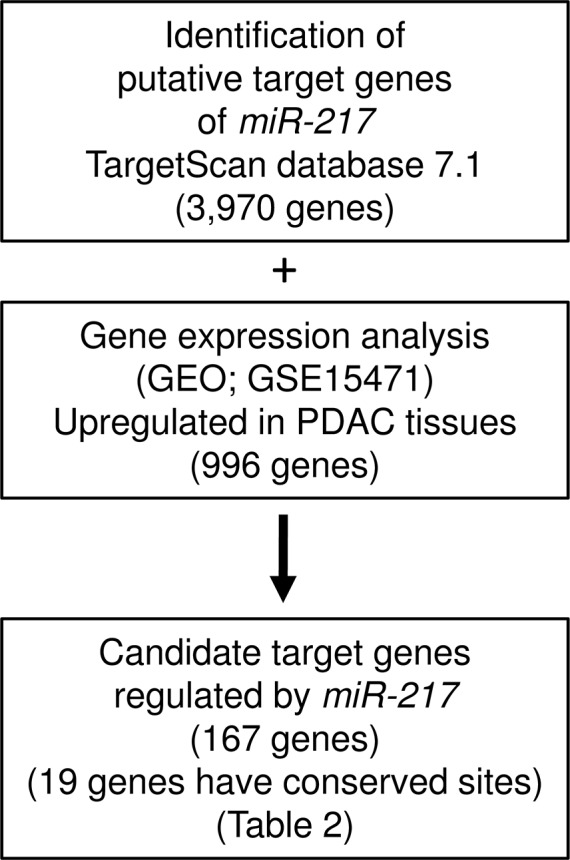
The strategy for analysis of *miR-217* target genes Flow chart illustrates the strategy for analysis of *miR-217* target genes in PDAC cells.

To gain further insight into the molecular mechanisms and pathways regulated by tumor-suppressive *miR-217* in PDAC cells, we used *in silico* analyses. The strategy for narrowing down the genes targeted by *miR-217* is shown in Figure 2. The TargetScan database showed that 3,970 genes have putative target sites for *miR-217* in their 3′-UTRs. Gene expression data showed that 996 genes were upregulated (fold-change log_2_ > 1.5) in cancer tissues by GEO database analyses (GEO accession number; GSE15471). We identified 167 genes that were putative targets of *miR-217* and were upregulated in PDAC specimens. Finally, we found that 19 genes had conserved sequences that were putatively targeted by *miR-217* (Table 2).

**Table 2:** Candidate target genes regulated by *miR-217*

Entrez gene	Gene symbol	Gene name	Conserved sites				GEO data	*TCGA_OncoLnc
			8mer sites	7mer-m8 sites	7mer-A1 sites	Total	FC(log2)	P-value
9644	*SH3PXD2A*	SH3 and PX domains 2A	1	1	1	3	1.631	0.3620
54443	*ANLN*	anillin, actin binding protein	1	1	0	2	1.729	0.0014
3624	*INHBA*	inhibin, beta A	0	1	0	1	5.190	0.0414
1295	*COL8A1*	collagen, type VIII, alpha 1	1	0	0	1	4.623	0.3640
2335	*FN1*	fibronectin 1	0	1	0	1	3.727	0.0950
860	*RUNX2*	runt-related transcription factor 2	1	0	0	1	2.932	0.4790
10085	*EDIL3*	EGF-like repeats and discoidin I-like domains 3	1	0	0	1	2.560	0.5300
6400	*SEL1L*	sel-1 suppressor of lin-12-like (C. elegans)	1	0	0	1	2.375	0.0950
22943	*DKK1*	dickkopf WNT signaling pathway inhibitor 1	0	0	1	1	2.303	0.0110
800	*CALD1*	caldesmon 1	0	0	1	1	2.067	0.5440
10630	*PDPN*	podoplanin	0	1	0	1	1.957	0.3500
79070	*KDELC1*	KDEL (Lys-Asp-Glu-Leu) containing 1	1	0	0	1	1.870	0.0969
55454	*CSGALNACT2*	chondroitin sulfateN-acetylgalactosaminyltransferase 2	0	1	0	1	1.781	0.6930
57181	*SLC39A10*	solute carrier family 39 (zinc transporter),member 10	0	1	0	1	1.770	0.0007
6091	*ROBO1*	roundabout, axon guidance receptor, homolog 1 (Drosophila)	0	0	1	1	1.769	0.8790
22856	*CHSY1*	chondroitin sulfate synthase 1	0	0	1	1	1.652	0.3330
23603	*CORO1C*	coronin, actin binding protein, 1C	0	0	1	1	1.566	0.0056
50515	*CHST11*	carbohydrate (chondroitin 4) sulfotransferase 11	1	0	0	1	1.559	0.0024
9444	*QKI*	QKI, KH domain containing, RNA binding	0	0	1	1	1.554	0.9170

* Kaplan-Meier analysis log-rank P-value < 0.05

Poor prognosis with a high expression

We also checked the expression status of those 19 genes and the clinical significance of PDAC by using the OncoLnc database (http://www.oncolnc.org/). Kaplan-Meier survival curves showed that high expression of 6 genes was associated with poor prognosis in PDAC by TCGA database searching (Table 2, Supplementary Figure 3). In this study, we focused on the actin binding protein anillin (ANLN) because past studies had indicated that actin binding proteins were involved in metastasis [23–26].

### *ANLN* is a direct target of *miR-217* in PDAC cells

**Figure 3:**
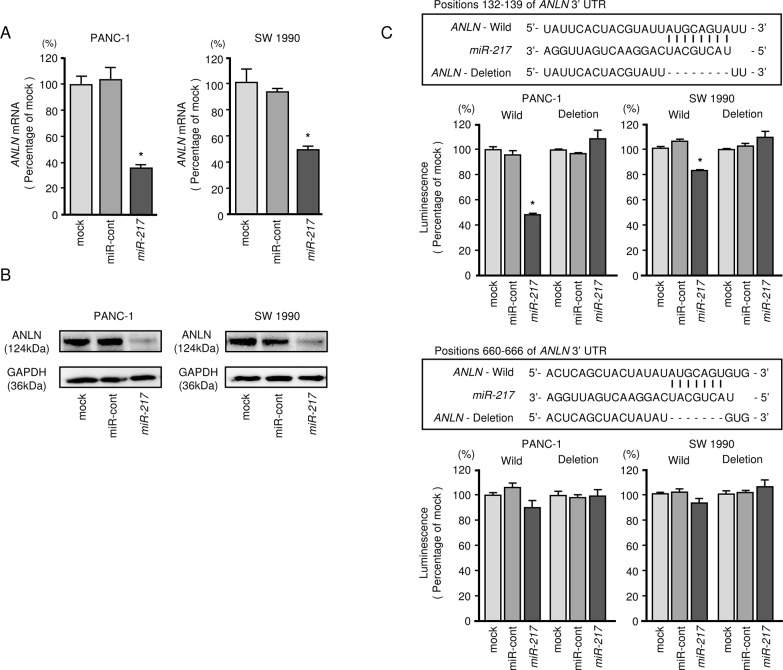
Direct regulation of *ANLN* by *miR-217* in PDAC cell lines **(A)** *ANLN* mRNA expression in PDAC cell lines was evaluated by qRT-PCR 72 h after transfection with *miR-217*. *GUSB* was used as an internal control. *, *P* < 0.0001. **(B)** ANLN protein expression in PDAC cell lines was evaluated by Western blot analyses 96 h after transfection with *miR-217*. GAPDH was used as a loading control. **(C)** *miR-217* binding sites in the 3′-UTR of *ANLN* mRNA. Dual luciferase reporter assays using vectors encoding putative *miR-217* (positions 132 - 139 or 660 - 666) target sites of the *ANLN* 3′-UTR for both wild-type and deleted regions. Normalized data were calculated as ratios of *Renilla*/firefly luciferase activities. *, *P* < 0.005.

We performed qRT-PCR to validate *miR-217* repression of *ANLN* mRNA expression in PDAC cell lines. Our studies revealed that *ANLN* mRNA was significantly reduced in *miR-217* transfectants in comparison with mock or miR-control transfectants (*P* < 0.0001 and *P* < 0.0001, Figure 3A). Expression of ANLN protein was also repressed in the *miR-217* transfectants (Figure 3B, Supplementary Figure 6).

Target prediction databases indicated two putative target sites in the 3′-UTR of *ANLN* (Figure 3C). To determine whether *ANLN* mRNA had a functional target site, we performed a dual luciferase reporter assay. We used vectors encoding the partial wild-type sequence of the3′-UTR of the mRNA, including the predicted *miR-217* target sites. We found that the luminescence intensity was significantly reduced by co-transfection with *miR-217* and the vector carrying the wild-type 3′-UTR (position 132 – 139), whereas transfection with the deletion vector (binding site had been removed) blocked the decrease in luminescence (*P* < 0.005, Figure 3C). In contrast, the luminescence intensity was not decreased in co-transfection with *miR-217* and the vector carrying the wild-type 3′-UTR (position 660 – 666) (Figure 3C).

### Effects of silencing *ANLN* on PDAC cell lines

**Figure 4:**
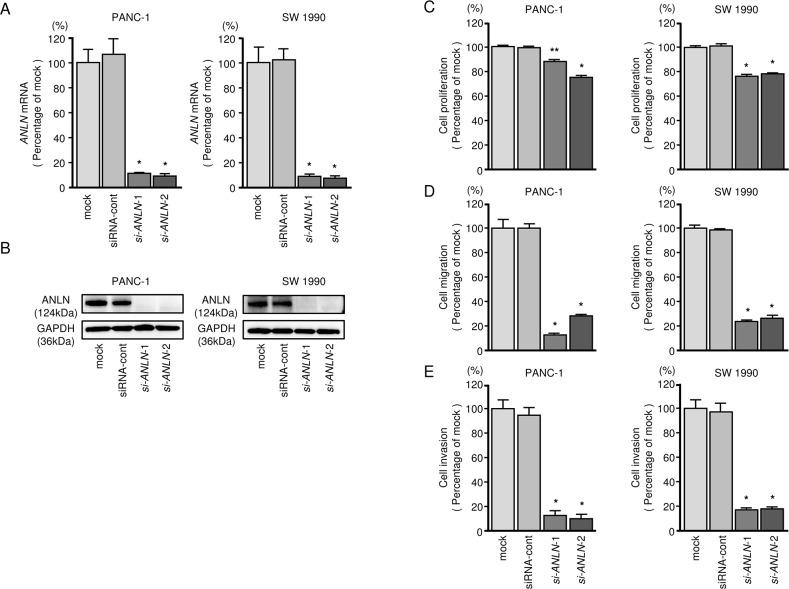
*ANLN* mRNA and ANLN protein expression after *si-ANLN* transfection and effects of *ANLN* silencing in PDAC cell lines **(A)** *ANLN* mRNA expression in PDAC cell lines was evaluated by qRT-PCR 72 h after transfection with *si-ANLN*-1 or *si-ANLN*-2. *GUSB* was used as an internal control. **(B)** ANLN protein expression in PDAC cell lines was evaluated by Western blot analysis 96 h after transfection with *miR-217*. GAPDH was used as a loading control. **(C)** Cell proliferation was determined with the XTT assays 72 h after transfection with 10 nM *si-ANLN*-1 or *si-ANLN*-2. *,*P* < 0.0001, **, *P* < 0.05. **(D)** Cell migration activity was determined by migration assays. *,*P* < 0.0001. **(E)** Cell invasion activity was determined using Matrigel invasion assays. *, *P* < 0.0001.

To investigate the functional role of A*NLN* in PDAC cells, we carried out loss-of-function studies using si-*ANLN* transfectants. First, we evaluated the knockdown efficiency of si-*ANLN* transfection in PDAC cell lines. In the present study, we used two types of si-*ANLN* (si-*ANLN-1* and si-*ANLN-2*). Based on qRT-PCR assessment and Western blot analyses, both siRNAs effectively downregulated *ANLN* expression in both cell lines (*P* < 0.0001, Figure 4A and 4B). XTT, cell migration and cell invasion assays demonstrated that cell proliferation, migration and invasion were inhibited in si-*ANLN* transfectants compared with mock- or siRNA-control-transfected cells (Figure 4C, 4D and 4E).

### Effects of cotransfection of *ANLN* and *miR-217* in PDAC cell line

To confirm the antitumor effect of *miR-217* by targeting *ANLN* in PDAC cells, we performed rescue experiments by *ANLN* overexpression in PANC-1 cells with *miR-217* restoration. The rescue studies indicated that cell migration and invasion properties were rescued by *ANLN* transfectants compared with cells with restored *miR-217* only (Supplementary Figure 4). These data indicated that *ANLN* expression was regulated by *miR-217* and expression of *miR-217* was induced antitumor effects of migration and invasion in PDAC cells.

### Expression of *ANLN* in PDAC clinical specimens and the TCGA database

**Figure 5:**
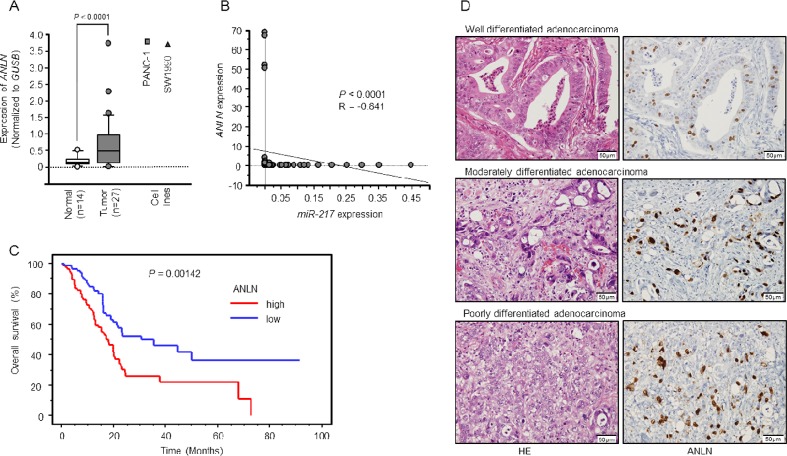
Expression levels of *ANLN* mRNA and immunohistochemical staining of ANLN protein in PDAC specimens **(A)** Expression levels of *ANLN* mRNA in PDAC or normal pancreatic tissues and PDAC cell lines. **(B)** The expression levels of *miR-217* and *ANLN* were negatively correlated (*R* = −0.841, P < 0.0001). **(C)** Kaplan–Meier curve analysis of overall patient survival between those with high *ANLN* expression and low *ANLN* expression in the PDAC TCGA database. **(D)** Immunohistochemical staining of ANLN in PDAC specimens. All differentiated types of PDAC (well, moderately, poorly) showed strong nuclear immunoreactivity (Left panel: hematoxylin-eosin staining, Right panel: ANLN staining, original magnification, X400).

*ANLN* was upregulated in clinical PDAC samples and cell lines, PANC-1 and SW1990 (*P* < 0.0001, Figure 5A). Negative correlations between *miR-217* expression and *ANLN* mRNA expression were found using Spearman's rank test (*R* = −0.841, *P* < 0.0001, Figure 5B).

Using a PDAC TCGA database, we found that the mRNA expression levels of *ANLN* were significantly upregulated. The *ANLN* high expression group had a significantly poorer overall survival by Kaplan-Meier analysis (Log-rank *P*-value = 0.00142, Figure 5C).

We confirmed the expression of ANLN protein in PDAC clinical specimens using immunohistochemistry. A total of 27 specimens were evaluated. All tumors were of the differentiated adenocarcinoma type (well, moderately, poorly) and showed strong nuclear immunoreactivity (Figure 5D).

### Investigation of downstream genes regulated by *ANLN* in PDAC cells

**Figure 6:**
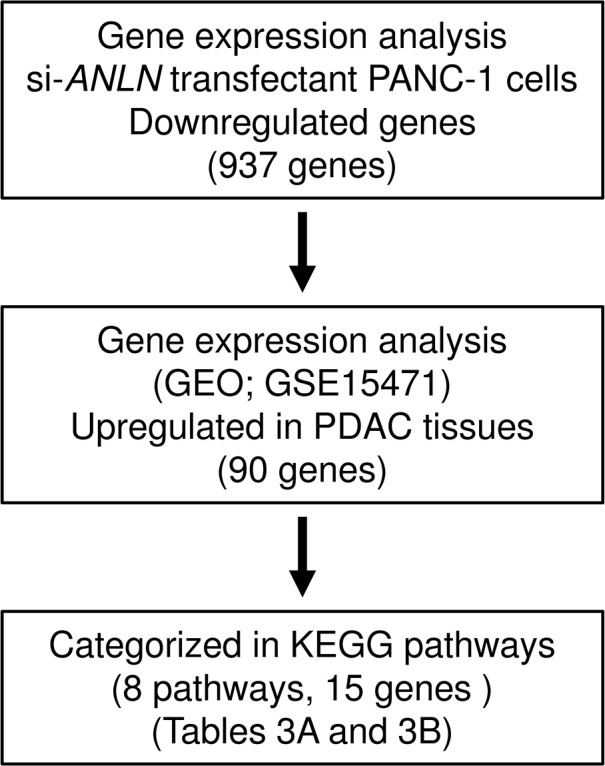
The strategy for analysis of *ANLN* downstream genes Flow chart illustrates the strategy for analysis of *ANLN*-mediated downstream pathways and genes in PDAC cells.

To identify the downstream genes regulated by *ANLN*, genome-wide gene expression and *in silico* analyses were performed in a PDAC cell line (PANC-1) transfected with si-*ANLN*. Our selection strategy is shown in Figure 6. A total of 937 genes were commonly downregulated (log_2_ ratio < - 1.0) in si-*ANLN-*transfected PANC-1 cells. We also assessed the downregulated genes using KEGG pathways and the GENECODIS program. With that approach, we identified 8 pathways and 15 genes that were significantly enriched (Table 3A and 3B). Our microarray expression data was deposited in Gene Expression Omnibus (GEO) database (accession number; GSE93290).

**Table 3A:** Enriched KEGG pathways in si-*ANLN* transfectant

KEGG ID	Pathways	P-value	No.of genes	Genes
Kegg:04510	Focal adhesion	0.00011	5	*ITGA3,RAC2,FLNA,LAMC2,MYL9*
Kegg:05200	Pathways in cancer	0.00105	5	*ITGA3,RAC2,FZD1,FZD2,LAMC2*
Kegg:04512	ECM-receptor interaction	0.00005	4	*ITGA3,HSPG2,LAMC2,CD47*
Kegg:04810	Regulation of actin cytoskeleton	0.00156	4	*ITGA3,RAC2,MSN,MYL9*
Kegg:04670	Leukocyte transendothelial migration	0.00013	3	*RAC2,MSN,MYL9*
Kegg:04610	Complement and coagulation cascades	0.00050	3	*F8,SERPINE1,PLAU*
Kegg:04310	Wnt signaling pathway	0.00050	3	*RAC2,FZD1,FZD2*
Kegg:05146	Amoebiasis	0.00186	3	*SERPINB9,LAMC2,GNA15*

**Table 3B:** Downregulated genes by si-*ANLN* transfectant in enrichied KEGG pathways

Entrez gene ID	Gene symbol	Gene name	*miR-217 target sites*	Expression FC(log2)		*TCGA_OncoLnc
				mock vs *si-ANLN*	GEO	P-value
8321	*FZD1*	frizzled class receptor 1	0	−1.603	1.186	0.2750
10398	*MYL9*	myosin, light chain 9, regulatory	0	−1.536	2.181	0.9090
961	*CD47*	CD47 molecule	0	−1.511	1.236	0.0705
5880	*RAC2*	ras-related C3 botulinum toxin substrate 2 (rho family, small GTP binding protein Rac2)	0	−1.444	1.323	0.7490
3918	*LAMC2*	laminin, gamma 2	0	−1.436	2.761	0.0595
3675	*ITGA3*	integrin, alpha 3 (antigen CD49C, alpha 3 subunit of VLA-3 receptor)	0	−1.363	1.329	0.0004
2316	*FLNA*	filamin A, alpha	0	−1.332	1.426	0.3290
2769	*GNA15*	guanine nucleotide binding protein (G protein), alpha 15 (Gq class)	0	−1.309	1.089	0.0070
5328	*PLAU*	plasminogen activator, urokinase	1	−1.252	2.341	0.0052
2535	*FZD2*	frizzled class receptor 2	0	−1.243	1.295	0.0349
3339	*HSPG2*	heparan sulfate proteoglycan 2	0	−1.177	1.080	0.3290
5272	*SERPINB9*	serpin peptidase inhibitor, clade B (ovalbumin), member 9	1	−1.111	1.670	0.3210
4478	*MSN*	moesin	1	−1.068	1.740	0.0389
5054	*SERPINE1*	serpin peptidase inhibitor, clade E (nexin, plasminogen activator inhibitor type 1), member 1	0	−1.060	1.966	0.0066
2157	*F8*	coagulation factor VIII, procoagulant component	0	−1.021	1.110	0.3080

* Kaplan-Meier analysis log-rank P-value < 0.05

Poor prognosis with a high expression

**Figure 7:**
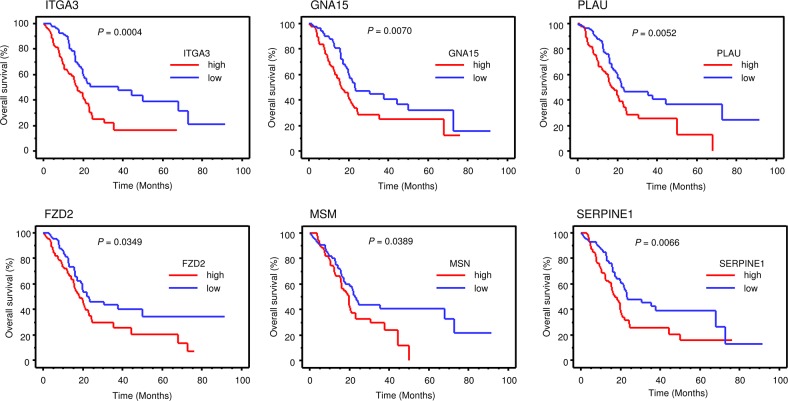
TCGA database analysis of *ANLN* downstream genes Kaplan–Meier plots overall survival with log-rank tests between those with high and low candidacy *ANLN* downstream 6 genes expression in the PDAC TCGA database.

Furthermore, we checked the expression status of those genes and their pathologic relations to PDAC by using the TCGA-based large cohort study data. Kaplan-Meier analysis showed that high expression group of 6 genes (*ITGA3*, *GNA15*, *PLAU*, *FZD2*, *MSM* and *SERPINE1*) had a significantly poorer overall survival for patients with PDAC (Table 3B and Figure 7).

## DISCUSSION

In this study, we confirmed the downregulation and antitumor function of *miR-217* in PDAC cells, showing that *miR-217* inhibited cancer cell migration and invasion. Based on those findings, we hypothesized that *miR-217* suppressed novel metastatic pathways in PDAC cells. In human genome, *miR-217* is located on the human chromosome 2q16.1 region and *miR-217* forms miRNA cluster with *miR-216s* (*miR-216a,miR-216b*). Expression of these clustered miRNAs were significantly downregulated in PDAC cells (data not shown). Past studies demonstrated that *miR-217* were downregulated in esophageal adenocarcinoma cells and leukemia cells [27, 28]. Moreover, our data showed that these miRNAs acted as antitumor miRNAs in PDAC cells (data not shown). First, we combined genome-wide gene expression analysis and *in silico* database search to identify antitumor *miR-217* targets in PDAC cells. Nineteen genes were identified as putative *miR-217* targets. Among them, we selected the actin-binding protein gene *ANLN* because our past studies showed that several actin-binding protein genes (*CORO1C*, *FSCN1*,*LASP1* and *MSN*) were overexpressed in cancer tissues and were deeply involved in promoting human cancer cell migration and invasion [29–32]. Interestingly, we showed that expression of these actin-binding proteins was regulated by several antitumor miRNAs (*miR-1*,*miR-133a*, *miR-145* and *miR-218*) [33–36]. Our present data demonstrated that *ANLN* was directly regulated by antitumor *miR-217* and knockdown of *ANLN* significantly inhibited cancer cell aggressiveness in PDAC cells.

The human *ANLN* gene was cloned as a human homologue of the *Anln* gene in *Drosophila melanogaster* that encodes a 1,125–amino acid actin-binding protein. ANLN has a unique multi-domain structure: an actin-binding and a myosin-binding domain at the N-terminus and a pleckstrin homology domain at the C-terminus [37]. ANLN is a conserved protein implicated in cytoskeletal dynamics, and it is a ubiquitously expressed protein required for cytokinesis. ANLN is primarily located in the nucleus in interphase, whereas in telophase it relocates to the cytoplasm where it accumulates in the contractile ring and cleavage furrow [38]. A small guanosine triphosphatase, RHOA, interacts with ANLN and stabilizes actin-related proteins (F-actin, myosin, septins and CD2AP) in the cleavage furrow [39].

Overexpressed *ANLN* was reported in several cancers and elevated expression appears to be involved in the metastatic potential of human cancers [23–25]. In non-small cell lung cancer (NSCLC), nuclear localization of ANLN was associated with poor survival of patients with NSCLC [40]. Likewise, detection of nuclear ANLN was significantly associated with decreased breast cancer survival and recurrence-free survival [41]. Our present immunohistochemical assessment of ANLN protein expression showed that ANLN was localized in cell nuclei in PDAC cells. Overexpression of *ANLN* was confirmed in PDAC cells, and Kaplan–Meier survival curves showed that high expression of *ANLN* predicted shorter survival in patients with PDAC by TCGA dataset analysis. Moreover, our data showed that reduced expression of *ANLN* in PDAC cells suppressed cancer cell migration and invasion. Past studies showed that knockdown of *ANLN* expression in breast cancer cells arrested the cell cycle in S/G2 or G2/M transition [26]. These findings suggest that (1) *ANLN* has multiple functions, (2) its expression affects several oncogenic pathways and (3) overexpression of *ANLN* enhances cancer cell aggressiveness.

To investigate the molecular pathways regulated by *ANLN* in PDAC cells, we applied a genome-wide gene expression analysis using si-*ANLN* transfectants. Our data showed that several pathways were downstream from *ANLN*, such as the “focal adhesion”, “pathways in cancer”, “ECM-receptor interaction” and “regulation of the actin cytoskeleton”. Finally, we identified 15 genes that were upregulated in PDAC specimens and involved in those pathways. Among the 15 genes, high expression of *ITGA3*, *GNA15*, *PLAU*, *FZD2*, *MSM* and *SERPINE1* predicted shorter survival in patients with PDAC as determined by TCGA database analysis. Our previous study showed that overexpression of *ITGA3* enhanced cancer cell migration and invasion and directly regulated antitumor expression of *miR-223* in prostate cancer [42].

Integrins play an important role in cell adhesion by linking the cytoskeleton of cells to components in the extracellular matrix (ECM) by integrin-mediated signaling pathways [43]. Several studies show that dysregulated ECM-integrin signaling is implicated in the development and progression of cancer [44, 45]. A complex of PLAUR and its ligand PLAU is an important regulator of ECM proteolysis, ECM interactions and cell signaling [46]. In cancer, aberrant expression of *PLAU* and *PLAUR* has been linked to tumor progression, metastasis, and shortened survival in cancer [47–49]. These findings suggest that *miR-217/ANLN-*mediated genes are deeply involved in PDAC pathogenesis. However, the detailed molecular mechanism how downstream genes of *ANLN* are regulated is still unclear. Future analysis is needed. Exploration of novel antitumor *miR-217*-mediated pathways may lead to the development of new treatment protocols for this disease.

In conclusion, downregulation of *miR-217* was detected in PDAC clinical specimens. *miR-217* acts as an antitumor miRNA through its targeting of *ANLN* expression in PDAC cells. Expression of *ANLN* enhanced cancer cell aggressiveness and its high expression predicts poorer survival of PDAC patients. Elucidation of *miR-217/ANLN*-mediated molecular networks may lead to a better understanding of PDAC pathogenesis and the development of new treatment protocols.

## MATERIALS AND METHODS

### Clinical specimens and cell lines

Clinical tissues specimens (n = 27) were collected from patients with PDAC who underwent curative surgical resection at Kagoshima University Hospital between 1997 and 2015. Normal pancreatic tissue specimens (n = 14) were obtained from noncancerous tumor-adjacent tissue. Each surgical specimen was histologically classified according to the TNM classification system [50]. All patients in this study provided informed consent and the study protocol was approved by the Institutional Review Board of Kagoshima University. Two human PDAC cell lines were investigated in this study. PANC-1 cells were obtained from RIKEN Cell Bank (Tsukuba, Ibaraki, Japan) and SW 1990 cells were obtained from the ATCC (Manassas, Virginia, USA).

Total RNA, including miRNA, was isolated using ISOGEN (NIPPON GENE, Toyama, Japan) according to the manufacturer's protocol.

### Quantitative real-time PCR (qRT-PCR)

Quantification of miRNA was performed using qRT-PCR as previously described [12, 13, 17, 51, 52]. Briefly, miRNAs were quantified using stem-loop RT-PCR, TaqMan MicroRNA Assays and Assay-on-Demand Gene Expression TaqMan probes and primers as directed by the manufacturer. Probes and primers for *miR-217* (product ID: 002337; Thermo Fisher Scientific, Waltham, MA, USA), *ANLN* (product ID: Hs01122612_m1; Thermo Fisher Scientif), human *GUSB* (product ID: Hs99999908_m1; Thermo Fisher Scientific) and *RNU48* (product ID: 001006; Thermo Fisher Scientific) were used as internal controls. Expression fold-changes were determined using the ΔΔCt method.

### Transfection of miRNA mimic and small interfering RNA (siRNA) into PDAC cell lines

PDAC cell lines were transfected with a miRNA mimic for gain-of-function experiments and siRNA for loss-of-function experiments. Pre-miR^TM^ miRNA precursors for *miR-217* (product ID: PM 12774), negative control miRNA (product ID: AM 17111), two *ANLN* siRNAs (product IDs: HSS122893 and HSS122895) and negative control siRNA (product ID: D-001810-10) were purchased from Thermo Fisher Scientific. The transfection efficiencies of miRNA in PANC-1 and SW 1990 cells were calculated as described in previous studies [12, 13, 17, 51, 52].

### Cell proliferation, migration and invasion assays

XTT assays were used to assess cell proliferation (Cell Proliferation Kit II, Roche Applied Sciences, Penzberg, Germany). Cell migration assays were performed with BD Falcon Cell Culture Inserts (BD Biosciences, Franklin Lakes, NJ, USA). Modified Boyden chambers containing Transwell-precoated Matrigel membrane filter inserts were used to quantitate cellular invasion. The protcols of these assays were described as previously [12, 13, 17, 51, 52].

### Western blot analyses

Protein lysates were collected 96 h after transfection and 20 μg of protein were separated using gel electrophoresis on e-PAGEL 5-20% gels (ATTO, Tokyo, Japan) before transfer to polyvinylidene fluoride membranes. Solutions of mouse anti-ANLN antibodies (product ID: AMAb90662; Atlas Antibodies AB, Stockholm, Sweden) were diluted 1:750 for immunoblotting. Anti-GAPDH antibodies at a 1:1000 dilution (product ID: SAF6698; Wako, Osaka, Japan) were used as an internal loading control. A detailed description of the Western blotting procedure was published elsewhere [12, 13, 17, 51, 52].

### Immunohistochemistry

Tissue sections were incubated overnight at room temperature with anti-ANLN antibodies diluted 1:200 (product ID: AMA90662; Atlas Antibodies AB, Stockholm, Sweden). Overnight, antibodies were visualized using an avidin-biotin complex (ABC) detection kit (Vector Laboratories, Burlingame, CA, USA) and a diaminobenzidine substrate system according to the manufacturer's protocol. The expression of *ANLN* was evaluated in cancer-rich fields using high-power microscopy (400×).

### Genome-wide gene expression and *in silico* analyses

To identify *miR-217* target genes, we used *in silico* analyses conducted as described previously [12, 13, 17, 51, 52]. The microarray data were deposited into the GEO database (accession number GSE15471). Next, we selected putative miRNA target genes using the TargetScanHuman 7.1(June, 2016 release, http://www.targetscan.org/vert_71) database and TCGA (The Cancer Genome Atlas, https://cancergenome.nih.gov/). Figure 2 shows the strategy for selecting target genes.

### TCGA database analysis of PDAC specimens

The clinical significance of *ANLN* in PDAC was assessed by RNA sequencing in TCGA OncoLnc (http://www.oncolnc.org/) [53, 54]. We linked TCGA survival data to mRNA expression levels. We obtained clinical information on 174 PDAC samples from the National Cancer Institute CDC Data Portal (https://gdc-portal.nci.nih.gov/projects/TCGA-PAAD). We selected high and low *ANLN* expression groups defined by the median value, and data were analyzed by Kaplan-Meier survival curves and log-rank statistics.

### Plasmid construction and dual-luciferase reporter assay

The partial wild-type sequence of the *ANLN* 3′-untranslated region (UTR) was inserted between the XhoI–PmeI restriction sites in the 3′-UTR of the *hRluc* gene in the psiCHECK-2 vector (C8021; Promega, Madison, WI, USA). Alternatively, we used sequences that were missing the *miR-217* target sites (position 132 - 139 or position 660 - 666). The synthesized DNA was cloned into the psiCHECK-2 vector. PANC-1 and SW1990 cells were transfected with 20 ng of the vector, 20 nM microRNAs, and 1 μL Lipofectamine 2000 (Invitrogen, Carlsbad, CA, USA) in 100 μL Opti-MEM (Invitrogen). The procedure of dual-luciferase reporter assay was described previously [12, 13, 17, 51, 52].

### Identification of downstream targets regulated by *ANLN* in PDAC

We used genome-wide gene expression analysis in a PDAC cell line (PANC-1) transfected with si-*ANLN*. Genes downregulated by *ANLN* were categorized by KEGG pathways using the GENECODIS program.

### Statistical analysis

The relationships between 2 groups and expression values obtained by RT-qPCR were analyzed using the Mann-Whitney U-test. The correlation between expression of *miR-217* and *ANLN* was evaluated using Spearman's rank test. The relationships among more than 3 variables and numerical values were analyzed using the Bonferroni-adjusted Mann-Whitney U-test. Overall survival (OS) after surgery was gauged using Kaplan–Meier curves. Patients were divided into two groups based on *ANLN* expression, and differences in survival were estimated using the log-rank test. We used Expert StatView software (version 5.0 SAS Institute Inc., Cary, NC, USA) for these analyses [12, 13, 17].

## SUPPLEMENTARY MATERIALS FIGURES AND TABLES


