# Supplementary material for: Regulation of actin-binding protein ANLN by antitumor miR-217 inhibits cancer cell aggressiveness in pancreatic ductal adenocarcinoma
Source: Oncotarget. 2017 May 29;8(32):53180–93. doi: 10.18632/oncotarget.18261 (PMC5581102; doi:10.18632/oncotarget.18261)
Supplement: Supplementary file 1 [file oncotarget-08-53180-s001.pdf]

Regulation of actin-binding protein ANLN by antitumor *miR-217* inhibits cancer cell aggressiveness in pancreatic ductal adenocarcinoma

SUPPLEMENTARY INFORMATION

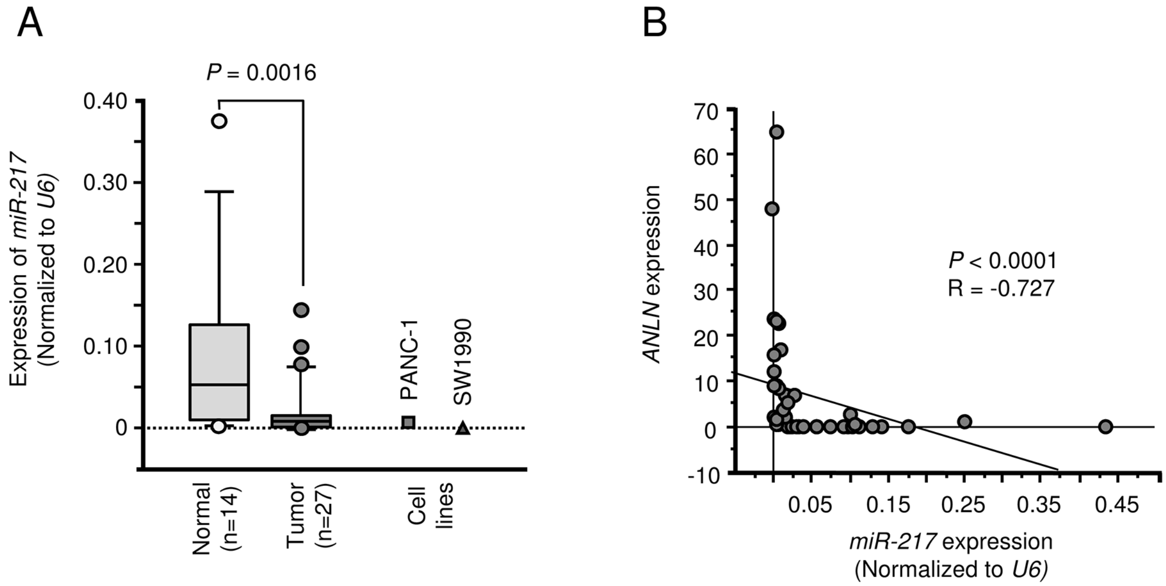

Supplementary Figure 1: Expression levels of *miR-217* on clinical samples and PDAC cell lines of normalized to *U6*. **(A)** Expression levels of *miR-217* in PDAC clinical specimens and cell lines were determined by qRT-PCR. Data were normalized to *U6* (product ID: 001973; Thermo Fisher Scientific) expression ($P = 0.0016$). **(B)** The expression levels of *miR-217* and *ANLN* were negatively correlated ($R = -0.727$, $P < 0.0001$). There was no actual differences between endogenous control *RNU48* and *U6* to low expression of *miR-217* in PDAC clinical samples and cell lines.

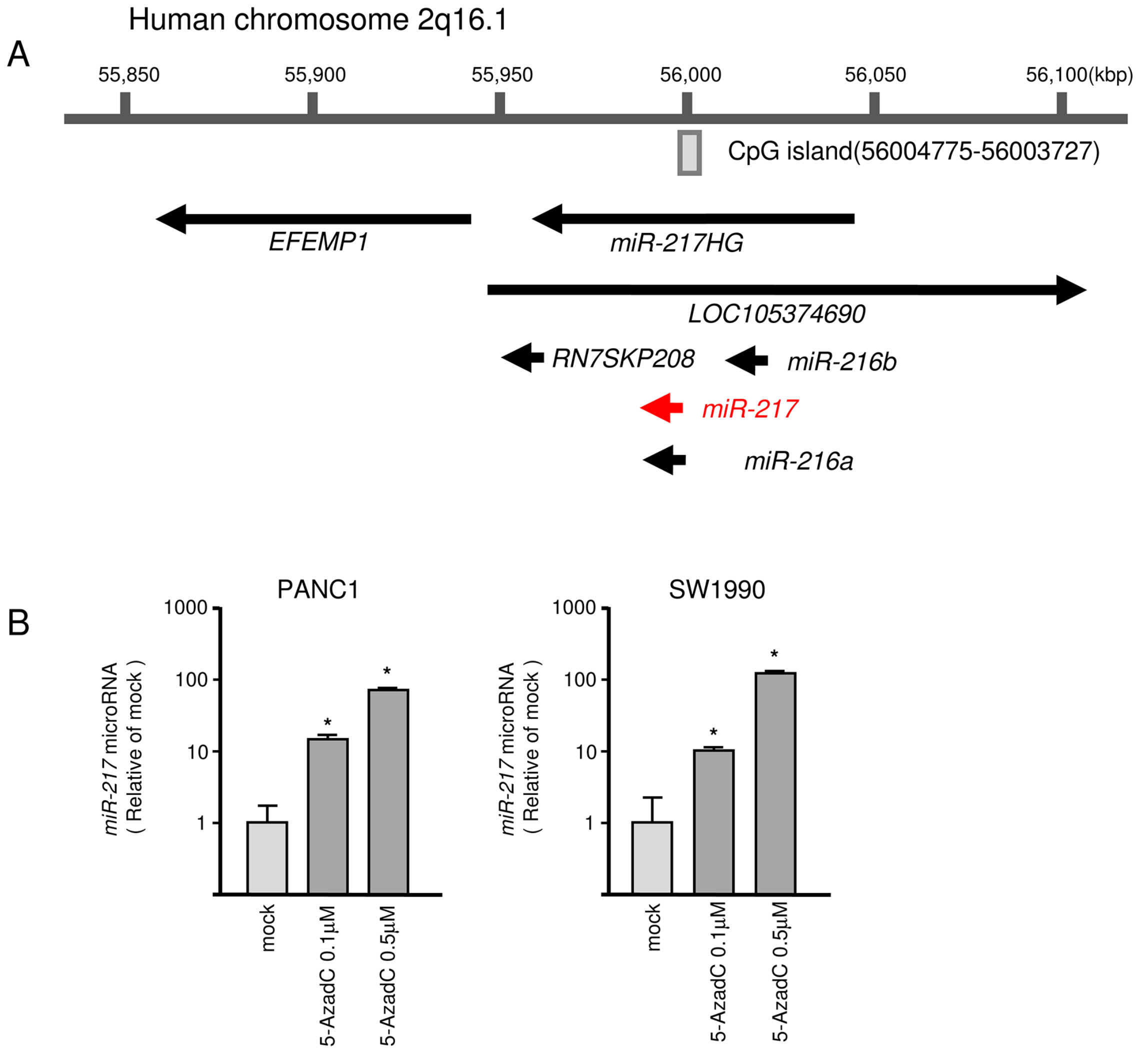

Supplementary Figure 2: Demethylation regulation induced high expression of *miR-217* in PDAC cell lines. (A) Genome map of human chromosome 2q16.1 region. The *miR-217, miR-216a* and *miR-216b* are encoded within cluster. CpG island is located on promoter region of *miR-217*. **(B)** Effect of demethylating agent [5-aza-2'-deoxycytidine (5-aza-dC)] (Wako, Osaka, Japan) 72 h treatment 0.1 µM and 0.5 µM on PDAC cell lines (PANC-1, SW1990). The expression level of *miR-217* was normalized to *RNU48* (*$P < 0.05$).

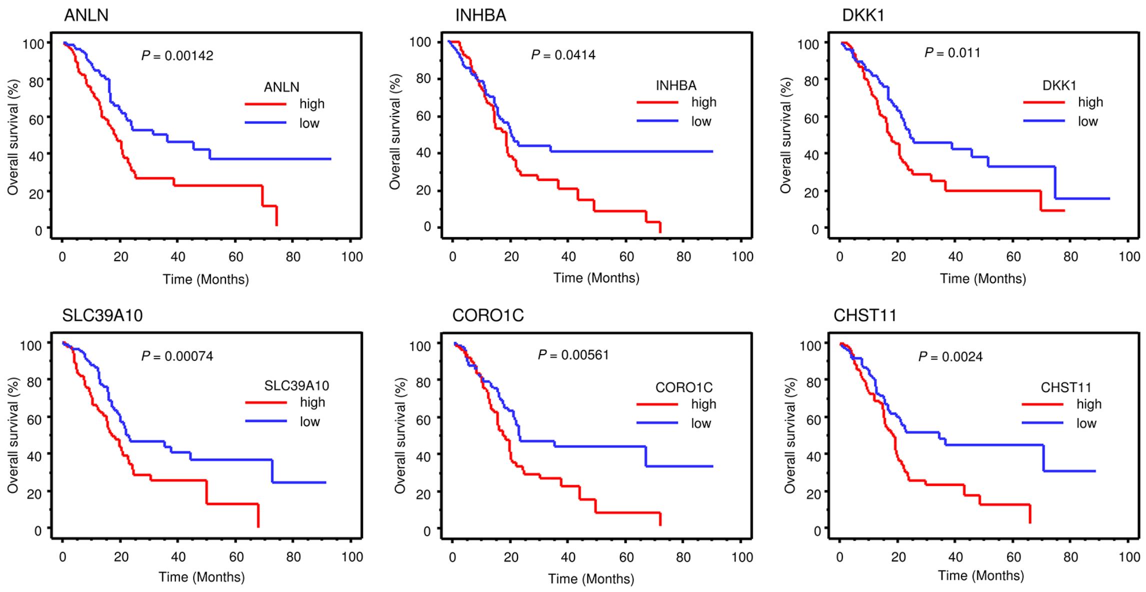

Supplementary Figure 3: TCGA database analysis of candidacy *miR-217* target genes. Kaplan–Meier plots overall survival with log-rank tests between those with high and low candidacy *miR-217* target 6 genes expression in the PDAC TCGA database.

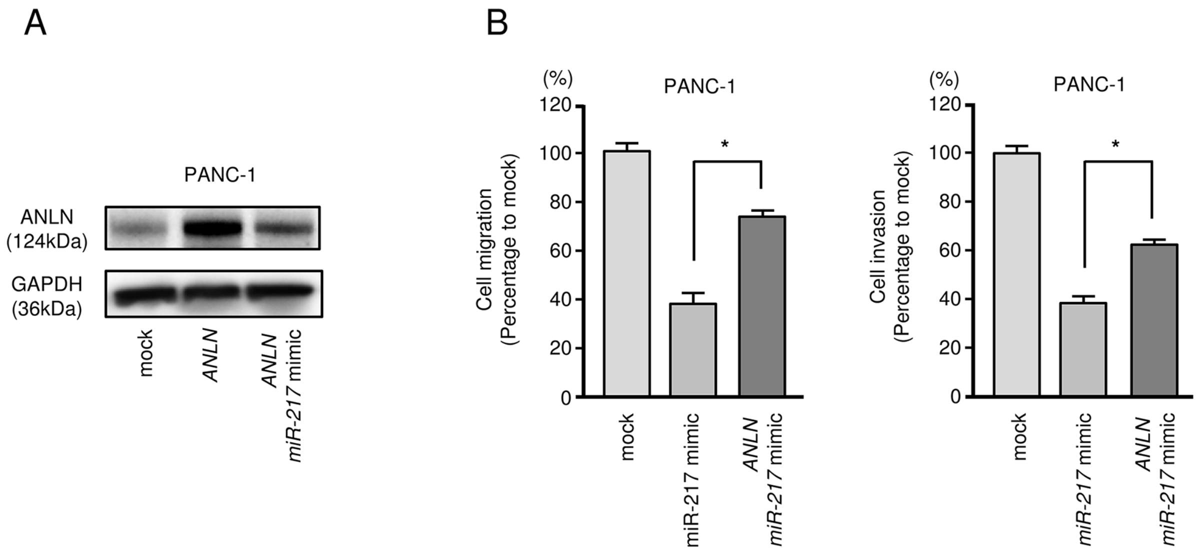

Supplementary Figure 4: Rescue experiments by inducing *ANLN* overexpression in PANC-1 with *miR-217* restoration. For *ANLN* overexpression studies, PDAC cell lines were transfected with pCMV6-vecter of *ANLN* cDNA clone (Origene Technologies, TrueORF Gold, Accession No: NM_018685) using Lipofectamine 3000 (Life Technologies). Cell culture of forward transfection used 6 well plates according to the protocol. **(A)** *ANLN* protein overexpression in PANC-1 was evaluated by Western blot analyses 72 h after 0.5 µg/well transfection with *ANLN* cDNA plasmid (middle) and *ANLN* protein overexpression was attenuation 72 h co-transfection with 0.5 µg/well *ANLN* cDNA plasmid and 10 µM *miR-217* mimic (right). GAPDH was used as a loading control. **(B)** Cell migration activity of 72 h co-transfection with 0.5 µg/well *ANLN* cDNA plasmid and 10 µM *miR-217* mimic was determined by migration assays. *, $P < 0.0001$. Cell invasion activity of 72 h co-transfection with 0.5 µg/well *ANLN* cDNA plasmid and 10 µM *miR-217* mimic was determined using Matrigel invasion assays. *, $P < 0.0001$.

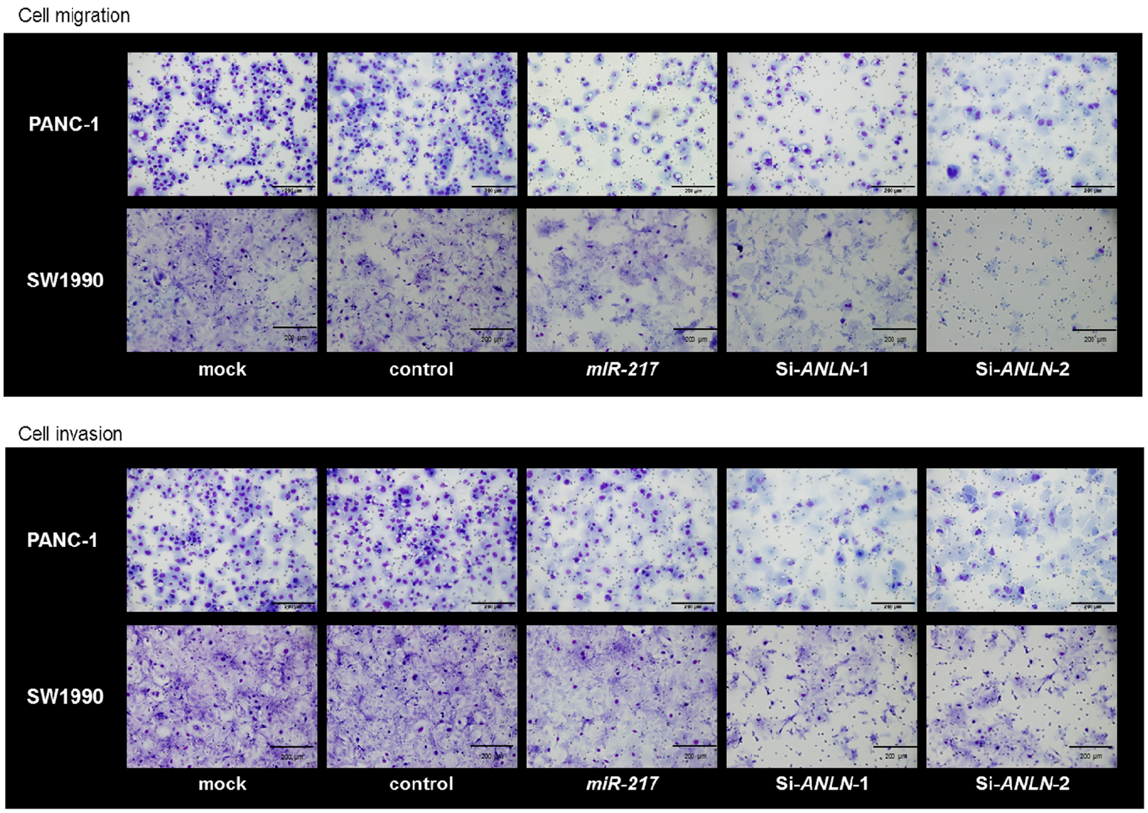

Supplementary Figure 5: Representative data of cell migration and invasion in PDAC cell lines. Photomicrograph of migration and invasion assays with transfection *miR-217* mimic and si-*ANLN*-1, si-*ANLN*-2 were presented antitumor function in PDAC cell lines.

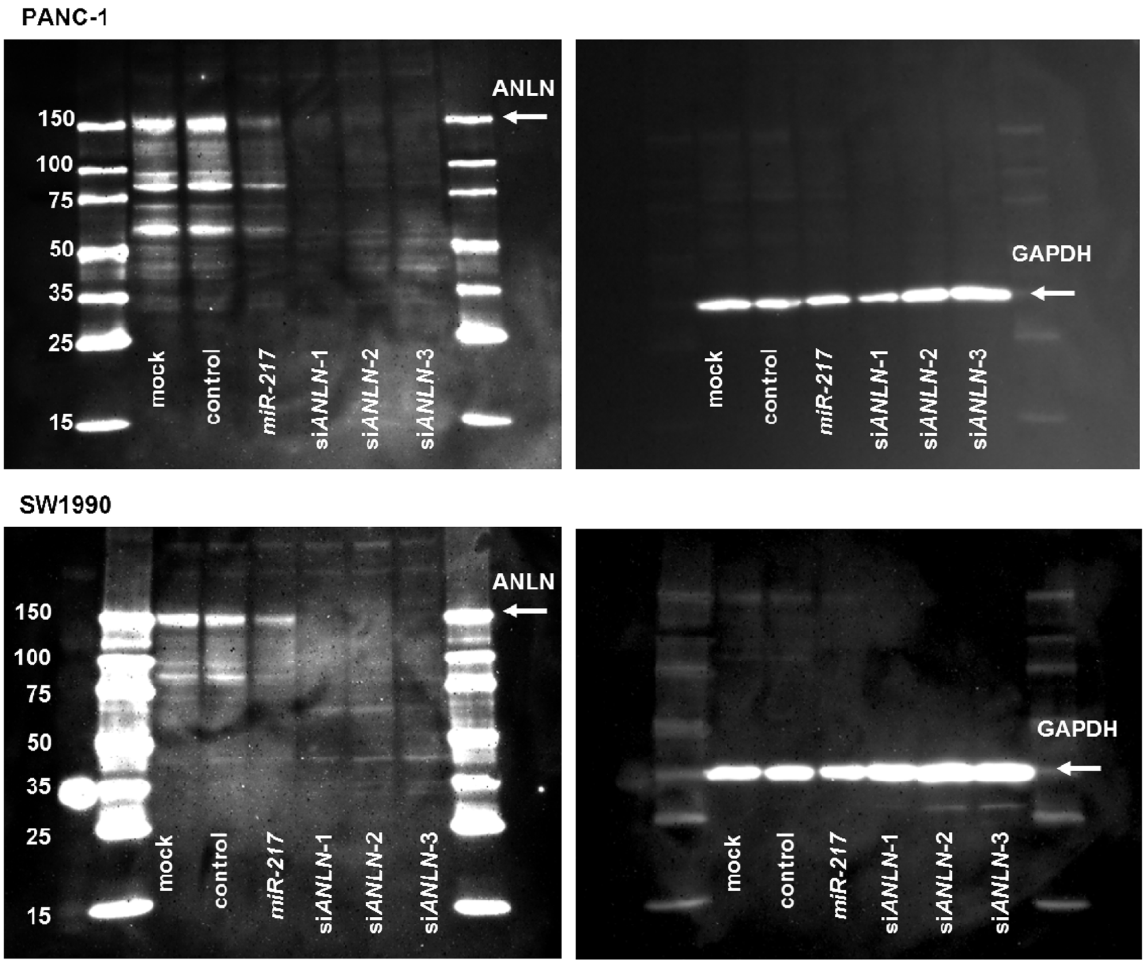

Supplementary Figure 6: Whole image of Western blotting in PDAC cell lines. Original data of Western blot analysis, ANLN protein expression in PDAC cell lines was evaluated transfection with *miR-217* and si-*ANLN*-1, si-*ANLN*-2. GAPDH was used as a loading control.
